# The global research of magnetic resonance imaging in Alzheimer’s disease: a bibliometric analysis from 2004 to 2023

**DOI:** 10.3389/fneur.2024.1510522

**Published:** 2025-01-15

**Authors:** Xiaoyu Sun, Jianghua Zhu, Ruowei Li, Yun Peng, Lianggeng Gong

**Affiliations:** ^1^Department of Radiology, The Second Affiliated Hospital, Jiangxi Medical College, Nanchang University, Nanchang, China; ^2^Jiangxi Provincial Key Laboratory of Intelligent Medical Imaging, Nanchang, China

**Keywords:** Alzheimer’s disease, magnetic resonance imaging, bibliometric, VOSviewer, CiteSpace

## Abstract

**Background:**

Alzheimer’s disease (AD) is a common neurodegenerative disorder worldwide and the using of magnetic resonance imaging (MRI) in the management of AD is increasing. The present study aims to summarize MRI in AD researches via bibliometric analysis and predict future research hotspots.

**Methods:**

We searched for records related to MRI studies in AD patients from 2004 to 2023 in the Web of Science Core Collection (WoSCC) database. CiteSpace was applied to analyze institutions, references and keywords. VOSviewer was used for the analysis of countries, authors and journals.

**Results:**

A total of 13,659 articles were obtained in this study. The number of published articles showed overall exponential growth from 2004 to 2023. The top country and institution were the United States and the University of California System, accounting for 40.30% and 9.88% of the total studies, respectively. Jack CR from the United States was the most productive author. The most productive journal was the Journal of Alzheimers Disease. Keyword burst analysis revealed that “machine learning” and “deep learning” were the keywords that frequently appeared in the past 6 years. Timeline views of the references revealed that “#0 tau pathology” and “#1 deep learning” are currently the latest research focuses.

**Conclusion:**

This study provides an in-depth overview of publications on MRI studies in AD. The United States is the leading country in this field with a concentration of highly productive researchers and high-level institutions. The current research hotspot is deep learning, which is being applied to develop noninvasive diagnosis and safer treatment of AD.

## 1 Introduction

Alzheimer’s disease (AD) is the most common neurodegenerative disease in elderly individuals and can cause progressive memory loss and cognitive impairment (1). It is considered a serious problem for both individual health and government healthcare systems worldwide (2). AD is characterized by the accumulation of *β*-amyloid (Aβ) plaques and neurofibrillary tangles (NFTs) composed of hyperphosphorylated tau protein in the brain, which impair neuronal function and communication (3, 4). The advent of new anti-amyloid monoclonal antibodies such as aducanemab, lecanemab, and donanemab as treatment for early AD, may slow disease progression but also pose significant risks such as amyloid related imaging abnormalities (ARIA) (5–8). Although many studies have explored the pathogenesis, diagnostics and treatment of AD, the underlying mechanisms are currently not well understood.

In recent years, with the rapid development of medical imaging technologies, magnetic resonance imaging (MRI) has been widely applied to investigate the pathological features of AD, which provides a new perspective for better understanding the pathogenesis of AD. Structural MRI-based measures of medial temporal lobe atrophy are regarded as valid markers in clinical AD diagnosis (9–11). Researchers have used MRI as a unique and noninvasive tool to monitor cortical gray matter loss (12–14), white matter lesions (15), and small vessel abnormalities (16) longitudinally in AD patients. Then multimodal imaging techniques, including MRI and positron emission computed tomography (PET) technology, are also widely used in the early diagnosis and prognosis prediction of AD clinically, and researchers aim to provide important imaging evidence from the aspects of structure (9, 10, 17, 18), function (19–23), metabolism (24), and biomarkers (1, 25–27). Moreover, with rapid advancements in high-field MRI in small animals, many studies have been conducted to explore the underlying mechanisms and drug development of AD in animal models using high-field MRI (22, 28–31). Although these MRI studies have enhanced our understanding of the imaging features and underlying mechanisms of AD, little attention has been given to the current research status, hotspots, and frontier trends in this field (32).

In recent years, bibliometric analysis has been widely used to explore the literature in specific research fields, which can quantitatively analyze and visualize the literature data and measure characteristics through various bibliometric tools (33–35), thus helping researchers quickly and accurately understand the research status, hotspots, and trends of this topic in the field. In this study, we conducted a bibliometric analysis of publications related to MRI studies in AD in the Web of Science Core Collection (WoSCC) between 2004 and 2023. This study aimed to explore the research status, hotspots, and frontier trends of MRI studies in AD over the past 20 decades, which may help new researchers better grasp future research interest.

## 2 Methods

### 2.1 Database

The WoSCC database was chosen as the data source. It is the most frequently used and acceptable database for researchers in a variety of fields.

### 2.2 Search strategy

**Figure 1:**
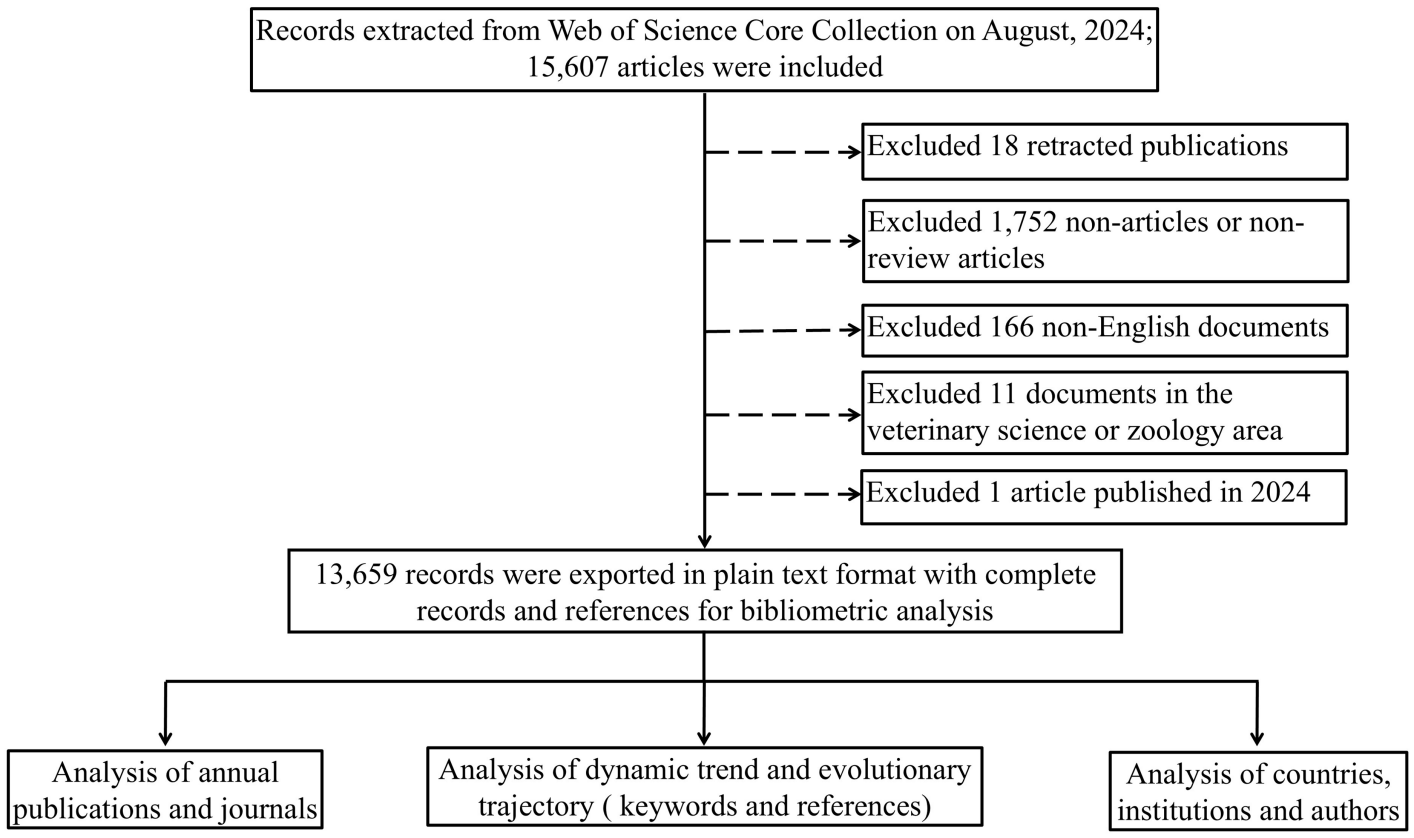
Work flow diagram of the study.

We searched for publications about MRI in the field of AD on August 26, 2024. The search query string was as follows: TS = (Alzheimer’s disease OR Alzheimer disease OR Alzheimer) AND (magnetic resonance imaging OR MRI OR T1WI OR T2WI OR DWI OR diffusion-weighted imaging OR ESWAN OR enhanced gradient echo T2 star weighted angiography OR SWI OR susceptibility weighted imaging OR MRS OR magnetic resonance spectroscopy OR ASL OR arterial spin labeling OR DCE OR dynamic contrast-enhanced OR PWI OR perfusion weighted imaging OR BOLD-fMRI OR blood oxygenation level-dependent functional magnetic resonance imaging OR DTI OR diffusion tensor imaging OR DKI OR diffusion kurtosis imaging OR IVIM OR intravoxel incoherent motion OR CEST OR chemical exchange saturation transfer OR APT OR amide proton transfer OR MPRAGE OR three-dimensional T1-weighted magnetization-prepared rapid gradient echo OR QSM OR quantitative susceptibility mapping OR rs-fMRI OR resting-state functional MRI OR fMRI OR functional magnetic resonance imaging OR VBM OR voxel-based morphology OR multimodal imaging technique OR volumetric MRI OR structural magnetic resonance imaging) AND (FPY = 2004–2023). The language was limited to English, and the document types were limited to articles and review articles. Information regarding titles, keywords, abstracts, authors, institutions and reference records of the papers was downloaded and saved in plain text format. The study flowchart is shown in Figure 1.

### 2.3 Data analysis and visualization

Microsoft Excel 2017 (Microsoft, Redmond, WA, United States) was used to display the global trend of publications and analyze the targeted files export statistical charts and tables of the most productive countries/regions, institutions, authors and journals.

VOSviewer (version 1.6.20) and CiteSpace (version 6.3. R1) are two widely used software programs for constructing and visualizing bibliometric networks (33, 36). CiteSpace was applied to analyze institutions, reference clusters and keyword bursts. VOSviewer was used for the analysis of countries/regions, authors and journals. The node in each map represents a country, institution or reference. The size of the node (country, institution or reference) represents the number of publications. The larger the node, the greater is the number of publications. The links between the nodes represent the strength of collaborations.

## 3 Results

### 3.1 Global trends in publications

**Figure 2:**
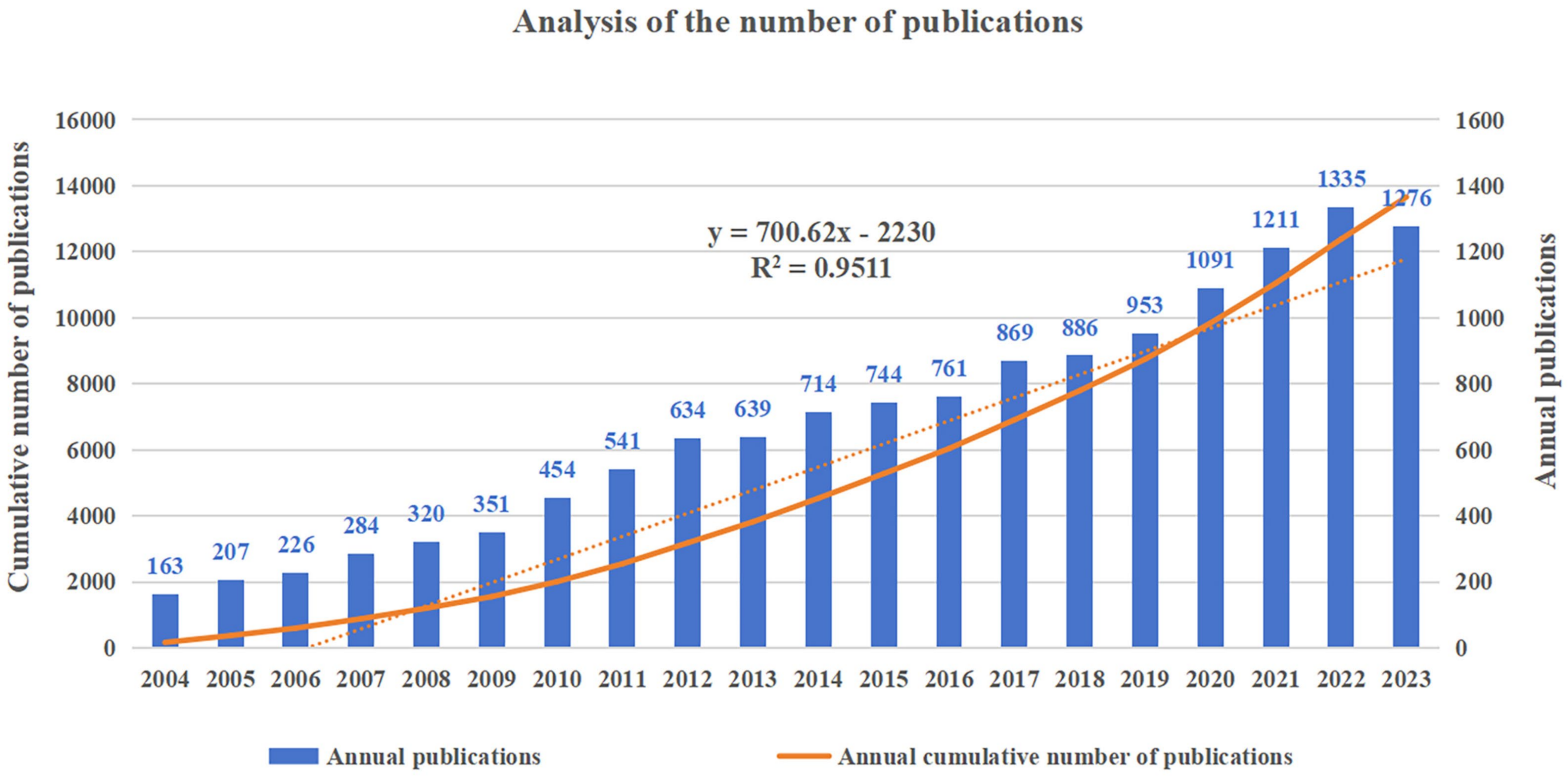
Global trend of publications on MRI research in AD from 2004 to 2023.

A total of 13,659 articles were included in the subsequent analyzes. The number of articles published each year and the cumulative number of articles published are shown in Figure 2, which shows a consistent overall upward trend in the number of annual publications from 2004 to 2022. The fastest annual growth was in 2020, with an increase of 138 articles. The statistical graph is adjusted to fit a linear curve that follows the following equation: the fitted curve index is *y* = 700.62x – 2230, with a correlation coefficient of 0.9511. In addition, the number of articles published in 2023 decreased from 1,335 in 2022 to 1,276, which may be caused by research bias in that it takes time for articles to reach a certain number of citations after publication.

### 3.2 Analysis of countries/regions and institutions

A total of 108 countries/regions published related articles in this field, 57 of which published no fewer than 10 articles. Table 1 shows the top 10 most productive countries/regions. The United States ranked first with 5,504 articles, accounting for 40.30% of the total number of articles published, followed by China (2,150, 15.74%), England (1,782, 13.05%), and Germany (1,192, 8.73%).

**Table 1:** Top 10 productive countries/regions.

Rank	Countries/Regions	Publications	Percentage	H-index
1	United States	5,504	40.30%	248
2	China	2,150	15.74%	104
3	England	1,782	13.05%	157
4	Germany	1,192	8.73%	119
5	Italy	930	6.81%	108
6	Canada	882	6.46%	109
7	Netherlands	845	6.19%	117
8	South Korea	791	5.79%	75
9	France	774	5.67%	107
10	Japan	731	5.35%	72

**Figure 3:**
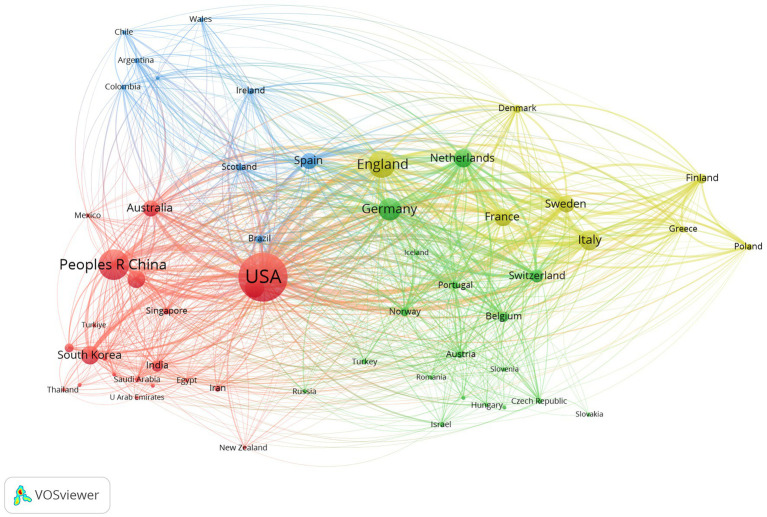
Country/region citation network visualization map generated via VOSviewer. The node represents a country/region, the size of the node represents the publication counts of a country/region, and the lines between nodes represent the strength of collaborations.

The H-index is a mixed index that is used as a significant indicator of the number and level of academic output of a scientific researcher, country, journal, or institution (37). The country with the highest H-index was the United States, followed by England, Germany and the Netherlands. A cooperative network of countries and their collaborations via VOSviewer is shown in Figure 3.

**Figure 4:**
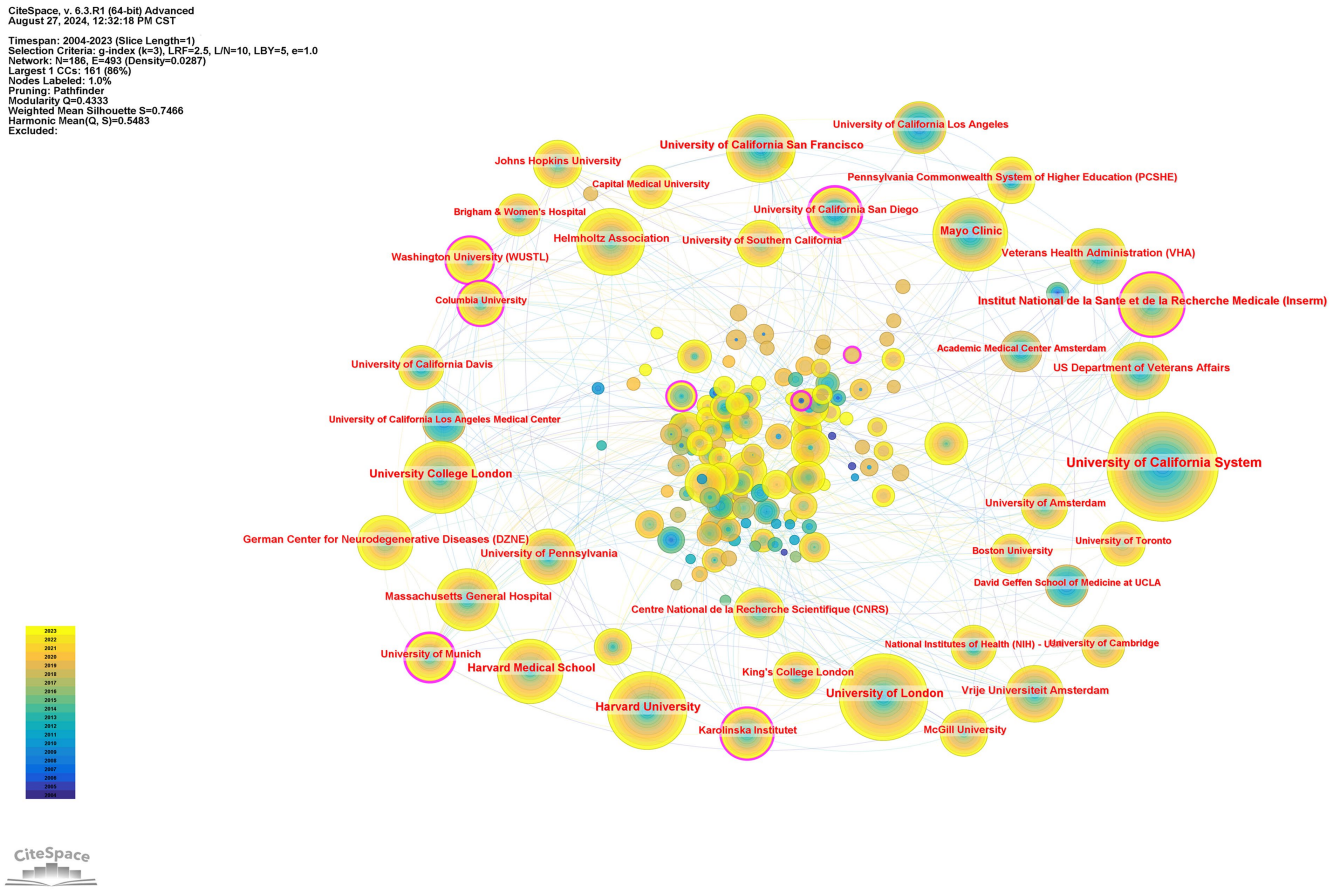
Institutions’ citation network visualization map generated via CiteSpace. The node represents an institution, the size of the node represents the publication counts of an institution, and the lines between nodes represent the strength of collaborations. The color of circles and the links between them reflect the occurrence time. The brighter they are, the more recently they occurred.

In addition, 9,233 institutions published articles in this field, of which 1,491 institutions had no fewer than 5 articles. Table 2 summarizes the top 10 institutions and their partnerships. The University of California System published the most, with 1,350 articles, accounting for 9.88%, followed by the University of London (854, 6.25%), Harvard University (738, 5.40%), and Mayo Clinic (625, 4.58%). The cooperative network of institutions and their collaborations via CiteSpace are shown in Figure 4.

**Table 2:** Top 10 productive institutions.

Rank	Institution	Publications	Percentage	H-index
1	University of California System	1,350	9.88%	163
2	University of London	854	6.25%	123
3	Harvard University	738	5.40%	126
4	Mayo Clinic	625	4.58%	130
5	University College London	625	4.58%	108
6	University of California San Francisco	548	4.01%	120
7	Harvard Medical School	523	3.83%	104
8	Institut National de la Sante et de Larecherche Medical Inserm	514	3.76%	90
9	Massachusetts General Hospital	494	3.62%	108
10	Helmholtz Association	477	3.49%	80

### 3.3 Analysis of authors

A total of 48,736 authors were included in this study, 3,784 of whom published no fewer than 5 articles. Table 3 summarizes the top 10 most productive authors. Half of the authors were from the United States. Among them, Jack CR, Scheltens P, and Petersen RC were the top 3 productive authors, with 413, 240, and 224 articles, respectively.

**Table 3:** Top 10 most productive authors.

Rank	Author	Country	Publications	H-index
1	Jack CR	United States	413	158
2	Scheltens P	Netherlands	240	149
3	Petersen RC	United States	224	179
4	Barkhof F	Netherlands	222	148
5	Weiner MW	United States	214	135
6	Knopman DS	United States	211	143
7	Moyer, Daniel	United States	189	147
8	Fox NC	United Kingdom	177	136
9	Van Der Flier WM	Netherlands	162	104
10	Teipel SJ	Germany	161	78

### 3.4 Analysis of journals

A total of 1,326 journals published related articles in this field, of which 367 authors published no fewer than 5 articles. The top 15 most prolific journals are listed in Table 4. Among the top 15 journals, one in three were from the United States. In addition, the journal with the highest IF was Brain among the top 15 journals. Journal of Alzheimers Disease [impact factor (IF) 2023: 3.4] published the most (1,175 publications), followed by Neurobiology of Aging (IF 2023: 3.7, 592 publications), and Neuroimage (IF 2023: 4.7, 561 publications). The top 15 journals published 5,460 articles, accounting for 40.12% of the total number of publications. These journals have made great contributions to the development of MRI studies in AD, indicating that more high-quality articles in this field will be published in these journals as a priority in the future.

**Table 4:** Top 15 productive journals according to the number of publications.

Rank	Journal	Country	Publications	JCR	IF (2023)
1	Journal of Alzheimers Disease	Netherlands	1,175	Q2	3.4
2	Neurobiology of Aging	England	592	Q2	3.7
3	Neuroimage	United States	561	Q1	4.7
4	Frontiers in Aging Neuroscience	Switzerland	513	Q2	4.1
5	Neurology	United States	404	Q1	7.7
6	Alzheimers and Dementia	United States	299	Q1	13
7	Neuroimage-Clinical	Netherlands	291	Q2	3.4
8	PLoS One	United States	287	Q1	2.9
9	Alzheimers Research and Therapy	United Kingdom	234	Q1	7.9
10	Brain	England	218	Q1	10.6
11	Human Brain Mapping	United States	209	Q1	3.5
12	Frontiers in Neuroscience	Switzerland	202	Q2	3.2
13	Current Alzheimer Research	Netherlands	169	Q4	1.8
14	Dementia and Geriatric Cognitive Disorders	Switzerland	164	Q4	1.4
15	Scientific Reports	England	162	Q1	3.8

### 3.5 Analysis of hotspots

#### 3.5.1 Analysis of keywords

**Figure 5:**
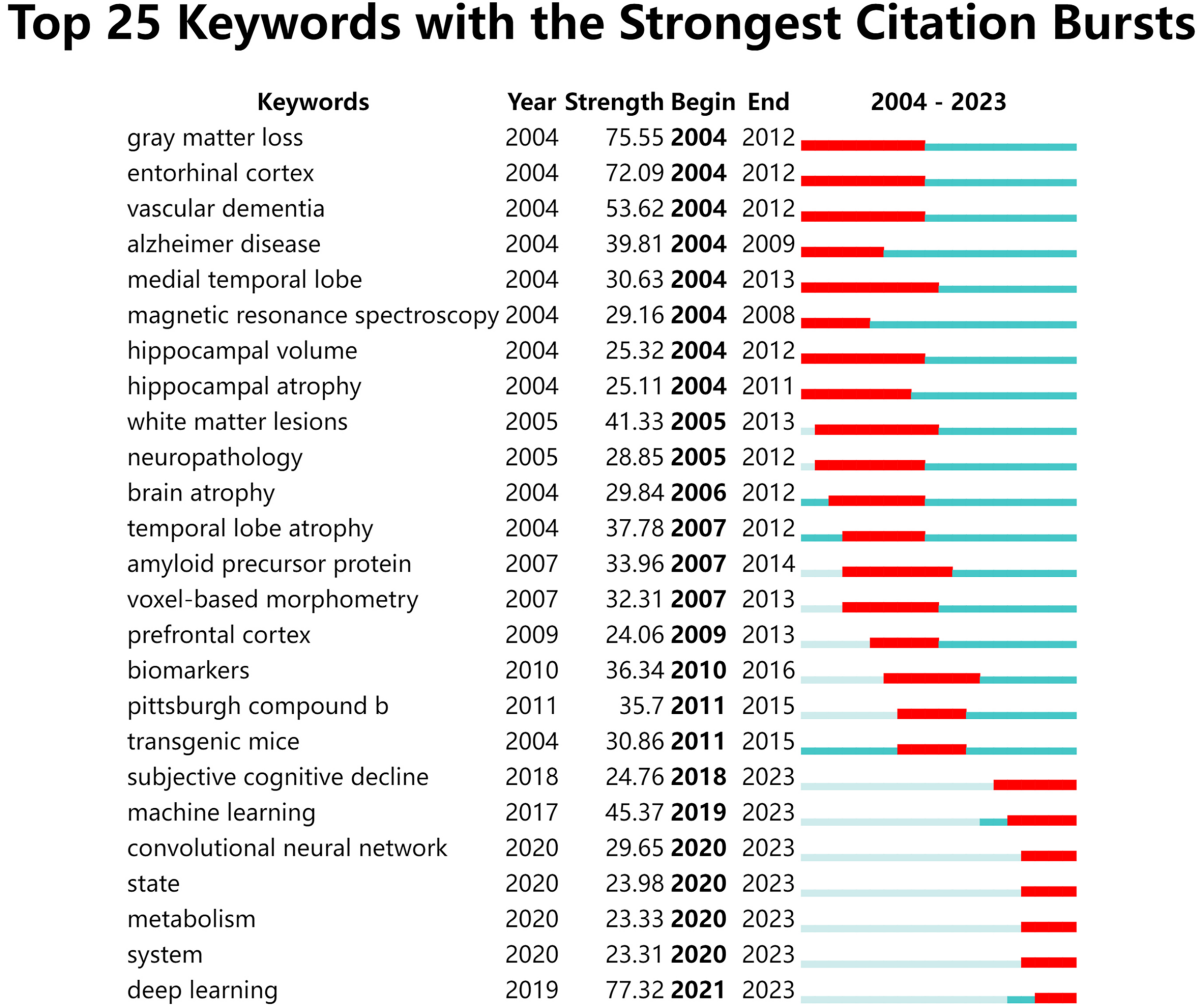
The top 25 keywords with the strongest citation bursts from 2004 to 2023 according to CiteSpace. The blue line represents the time period, and the red line represents the time span of the burst.

Keywords are the central ideas of an article. The research hotspots in MRI research on AD have been investigated mainly through keyword burst detection. The top 25 keywords according to CiteSpace are presented in Figure 5 and are sorted by the initial year of the burst. As shown in the diagram, “medial temporal lobe,” “entorhinal cortex,” “temporal lobe atrophy,” “gray matter loss,” “white matter lesions” and “hippocampal volume” had the longest burst durations (8–9 years), indicating the importance of these lesion locations for research in this field. “Deep learning,” “gray matter loss,” “entorhinal cortex,” and “vascular dementia” had the highest burst intensities, with burst strengths of 77.32, 75.55, 72.09, and 53.62, respectively. Moreover, “subjective cognitive decline,” “machine learning,” “convolutional neural networks” and “deep learning” appeared most frequently in the past 6 years, and they all lasted to the present, indicating that these are current and future research hotspots.

#### 3.5.2 Reference analysis

Reference analysis uses reference as the element of analysis to reflect the relationship between the references by analyzing patterns and trends in citations. CiteSpace software was used to build reference clustering. Each cluster was considered to represent a research focus.

From the analysis results, the modularity Q was 0.781, and the mean silhouette S was as high as 0.9202, indicating an excellent clustering effect and good network homogeneity.

**Figure 6:**
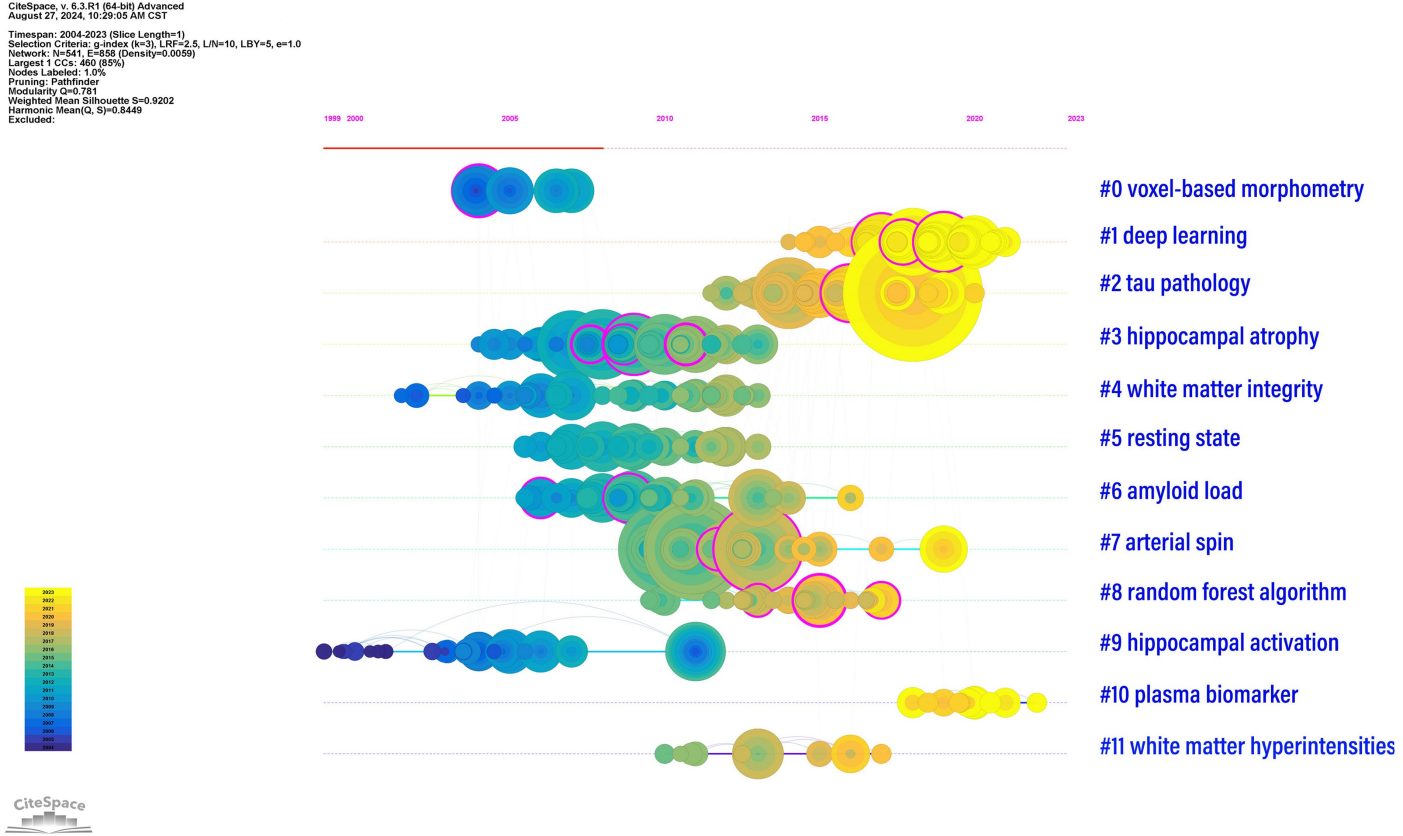
Timeline graph of reference clusters by CiteSpace. The node represents a reference, and the size of each node is associated with the number of references. Each line represents a cluster, and the numbers 0–11 refer to the top 12 clusters which represent 12 different research directions. The emergence time point and time span of all clusters are shown. Colors indicate the occurrence time: the brighter they are, the more recently they occurred.

Table 5 and Figure 6 present the 12 main clusters and their respective first five feature words. According to the clustering results, there were 12 clusters as follows: “#0 voxel-based morphometry,” “#1 deep learning,” “#2 tau pathology,” “#3 hippocampal atrophy,” “#4 white matter integrity,” “#5 resting state,” “#6 amyloid load,” “#7 arterial spin,” “#8 random forest algorithm,” “#9 hippocampal activation,” “#10 plasma biomarker,” and “#11 white matter hyperintensities.”

**Table 5:** Reference cluster by CiteSpace.

ClusterID	Size	Silhouette	Label (LLR)
0	69	0.955	Voxel-based morphometry (2992.45, 1.0E-4); using voxel-based morphometry (1914.88, 1.0E-4); whole-brain atrophy rate (1438.5, 1.0E-4); volumetric MRI study (1330.05, 1.0E-4); cingulate gyrus (1149.32, 1.0E-4)
1	60	0.982	Deep learning (5967.06, 1.0E-4); convolutional neural network (4964.83, 1.0E-4); artificial intelligence (3307.65, 1.0E-4); neural network (3216.05, 1.0E-4); using structural MRI (3201.32, 1.0E-4)
2	59	0.851	Tau pathology (6786.06, 1.0E-4); tau PET (5047.84, 1.0E-4); tau accumulation (4851.45, 1.0E-4); tau deposition (4281.51, 1.0E-4); subjective cognitive decline (4158.91, 1.0E-4)
3	59	0.922	Hippocampal atrophy (2054.71, 1.0E-4); ADNI cohort (2035.3, 1.0E-4); tensor-based morphometry (1790.55, 1.0E-4); multitask learning (1674.93, 1.0E-4); automated 3D mapping (1674.93, 1.0E-4)
4	40	0.908	White matter integrity (3551.81, 1.0E-4); white matter degeneration (3424.05, 1.0E-4); white matter microstructure (2253.75, 1.0E-4); uncinate fasciculus (1911.3, 1.0E-4); white matter disruption (1842.68, 1.0E-4)
5	32	0.956	Resting state (3769.44, 1.0E-4); default mode network (2944.09, 1.0E-4); default-mode network (1770.92, 1.0E-4); functional connectivity (1486.72, 1.0E-4); functional connection (1381.02, 1.0E-4)
6	31	0.843	Amyloid load (929.77, 1.0E-4); 42 measure (832.49, 1.0E-4); transforming cerebrospinal fluid (832.49, 1.0E-4); calculated pittsburgh compound B unit (832.49, 1.0E-4); dynamic biomarker (820.8, 1.0E-4)
7	30	0.879	Arterial spin (2945.98, 1.0E-4); diagnostic criteria (2619.73, 1.0E-4); cerebrospinal fluid (1991.38, 1.0E-4); prospective cohort study (1977, 1.0E-4); cerebral blood flow (1896.79, 1.0E-4)
8	30	0.839	Random forest algorithm (1340.56, 1.0E-4); feature extraction method (878.77, 1.0E-4); grading biomarker (871.55, 1.0E-4); major brain diseases (864.39, 1.0E-4); 5-year trend (864.39, 1.0E-4)
9	29	0.979	Hippocampal activation (1770.17, 1.0E-4); functional MRI studies (1474.67, 1.0E-4); parietal deactivation (1397.54, 1.0E-4); functional alteration (1230.53, 1.0E-4); genetic risk (1189.52, 1.0E-4)
10	12	1	Plasma biomarker (673.5, 1.0E-4); dementia risk (672, 1.0E-4); prognostic capabilities (541.39, 1.0E-4); diagnosing dementia (531.4, 1.0E-4); tau PET tracer (531.4, 1.0E-4)
11	9	0.992	White matter hyperintensities (1952.04, 1.0E-4); white matter lesion (1247.47, 1.0E-4); cerebral amyloid angiopathy (1127.61, 1.0E-4); age-associated aggregation (610.06, 1.0E-4); at-risk cohort (610.06, 1.0E-4)

As shown in Table 5, cluster #0 contained 69 keywords with the first five feature words of voxel-based morphometry, which included voxel-based morphometry, the whole-brain atrophy rate, volumetric MRI, and the cingulate gyrus. Cluster #1 included 60 keywords, and the first five keywords were deep learning, convolutional neural network, artificial intelligence, neural network, and structural MRI. Cluster #2 included 59 keywords, and the first five features were tau pathology, tau PET, tau accumulation, tau deposition, and subjective cognitive decline. Cluster #3 consisted of 59 keywords with the first five feature words of hippocampal atrophy, the ADNI cohort, tensor-based morphometry, multitask learning, and automated 3D mapping. Cluster #4 had 40 keywords, and the first five words were white matter integrity, white matter degeneration, white matter microstructure, uncinate fasciculus, and white matter disruption. Cluster #5 had 32 keywords, and the first five words were resting state, default mode network, default mode network, functional connectivity, and functional connection. Cluster #6 involved 31 keywords, with the first five feature words being amyloid load, 42 measure, transforming cerebrospinal fluid, calculated Pittsburgh compound B unit, and dynamic biomarker. Cluster #7 had 30 keywords, and the first five feature words extracted were arterial spin, diagnostic criteria, cerebrospinal fluid, prospective cohort study, and cerebral blood flow. Cluster #8 contained 30 keywords, with the first five words being the random forest algorithm, feature extraction method, grading biomarker, major brain diseases, and 5-year trend. Cluster #9 had 29 keywords, and the first five words were related to hippocampal activation, functional MRI studies, parietal deactivation, functional alteration, and genetic risk. Cluster #10 included 12 keywords, and the first five features were plasma biomarkers, dementia risk, prognostic capabilities, diagnosing dementia, and tau PET tracers. Cluster #11 involved 9 keywords, with the first five feature words being white matter hyperintensities, white matter lesions, cerebral amyloid angiopathy, age-associated aggregation, and at-risk.

In Figure 6, the above 12 clusters are displayed according to the reference time. Each line represents a cluster, where the nodes represent a reference, and the size of each node is associated with the number of references. Therefore, a timeline view of the references visually presents the phased hotspots of MRI studies in AD from the time dimension. As shown in Figure 6, “#0 voxel-based morphometry” and “#9 hippocampal activation” were the earliest studies in this field. “#0 tau pathology” and “#1 deep learning” are currently the latest research hotspots, suggesting that an increasing number of researchers are paying attention to the application of deep learning and tau pathology in MRI studies of AD.

## 4 Discussion

This study investigated the global trend of publications by authors from different countries and institutions, as well as the references and keywords in MRI studies on AD through bibliometric analysis from 2004 to 2023. The increasing number of annual publications suggests that an increasing number of researchers are paying attention to this field. It can be expected that the number of publications in this field will continue to remain high over the next few years. The most productive country was the United States. Jack CR from the United States was the most productive author. The most productive journal was the Journal of Alzheimers Disease, and the 15 journal with the highest IF was Brain. According to hotspot analysis, the period of the last 20 years can be roughly divided into 3 stages with different research hotspots, which sequentially evolved from (I) exploring the morphological changes in the AD brain by structural MRI (sMRI) to (II) identifying different biomarkers of AD and exploring the functional changes via functional MRI (fMRI) in AD patients and finally to (III) artificial intelligence (AI) applications.

In the current bibliographic study, CiteSpace software was used to construct a timeline graph of reference clusters and explore keyword bursts. According to the reference clustering results and keyword burst analysis, the evolution of hotspots in MRI studies of AD from 2004 to 2023 can be divided into three stages: stage I (2004–2010), stage II (2010–2018), and stage III (2018–2023).

### 4.1 Stage I

Stage I focused on the study of morphological changes in the AD brain via sMRI. The main keywords were associated with gray matter loss, the entorhinal cortex, temporal lobe atrophy, hippocampal atrophy, brain atrophy, and white matter lesions.

sMRI is widely used to explore the underlying pathophysiology of AD (38). Brain atrophy detected by sMRI is a valid biomarker of the stage and intensity of AD pathology (9, 10, 39). Structural changes in the brain map accurately upstream to Braak stages of NFT deposition (4, 40, 41) and downstream of neuropsychological deficits (42, 43). Considerable evidence suggests that AD initiates in the entorhinal cortex and hippocampus and spreads thereafter to the rest of the brain. The earliest sites of tau deposition and MRI-based atrophy are located along the perforant (polysynaptic) hippocampal pathway (entorhinal cortex, hippocampus and posterior cingulate cortex), which is consistent with early memory deficits (9, 44, 45). Progressive atrophy in the temporal, parietal and frontal neocortex is closely related to neuronal loss, as well as language, praxic, visuospatial and behavioral impairments (4, 17, 46, 47).

Several different processing methods, such as voxel-based morphometry (VBM) (48), boundary shift integral (BSI) (4, 49), and tensor-based morphometry (TBM) (45, 50), are employed to detect subtle changes and assess atrophy in the brain MR images of the same individual or groupwise comparisons. VBM studies have revealed that regional gray matter atrophy lies mainly in the bilateral mesial temporal lobes, including the hippocampus, amygdala, entorhinal cortex, and posterior cingulum, and extends to the frontal and parietal lobes with the progression of AD (51–53). VBM studies have also revealed white matter abnormalities in patients with AD compared with healthy controls (54–59). Nevertheless, structural neuroimaging studies of white matter volume in AD patients have yielded variable findings owing to heterogeneous subjects or small sample sizes, as well as methodological differences among studies. For example, two studies (56, 57) reported white matter volume reduction only in bilateral temporal structures, whereas other studies (54, 55, 58, 59) reported widespread white matter volume abnormalities beyond the temporal lobe. Li reported that white matter atrophy in AD patients occurred mainly in bilateral structures close to memory formations, such as the hippocampus, amygdala, and entorhinal cortex, through meta-analysis (60).

### 4.2 Stage II

Stage II focused on the study of biomarkers and fMRI in AD, and the main keywords were biomarkers, transgenic mice, and Pittsburgh compound b.

The major biomarkers of AD that are typically considered for clinical trials and observational studies are cerebrospinal fluid (CSF) Aβ1-42, CSF total-tau (t-tau), fluoro-deoxy-glucose positron emission tomography (FDG–PET), Pittsburgh compound B-PET (PIB–PET), and sMRI. As suggested by Wahlund and Blennow (61, 62), CSF tau, p-tau, and sMRI may reflect the disease stage or intensity of AD, whereas CSF Aβ represents a specific molecular pathway or etiology. These main CSF biomarkers have high diagnostic accuracy for identifying prodromal AD in the mild cognitive impairment (MCI) stage, with a sensitivity and specificity of 85–90% (25, 26). However, variability in measurements between clinical laboratories has hindered the identification of a unified critical value for CSF biomarkers because of differences in analytical procedures for manual ELISA methods between different laboratories, as well as variability in reagent quality and manufacturing procedures resulting in batch-to-batch variations (63). Research based on Alzheimer’s Disease Neuroimaging Initiative (ADNI) data has shown that sMRI is more closely related to cognition than CSF biomarkers are (64, 65).

In earlier stages of AD, such as MCI or preclinical AD, atrophy can be minimal, although metabolic imaging (FDG PET) findings may already be abnormal (66, 67). Although there are many amyloid imaging PET tracers based on ^11^C and ^18^F, the most studied tracer in the field of AD is PIB (68). C-pPIB can be used as a powerful biomarker of rCBF to measure neural activity and improve the diagnostic ability of PET for AD in conjunction with [^11^C]-aPIB (69). Tiepolt (70) reported that early [^18^F]FBB and [^11^C]PiB PET brain images are analogous to [^18^F]FDG PET images of the AD brain and that these tracers could be used as potential biomarkers in place of [^18^F]FDG. Furthermore, [^11^C]-PIB R (1) could serve as a complementary biomarker of neuronal activity and neurodegeneration in addition to the Aβ load given by [^11^C]-PIB BP(ND) (71). These findings suggest that Aβ deposition measured by PIB is an upstream process, whereas neurodegeneration is a downstream process initiated by Aβ deposition and is more closely related to cognitive decline (10, 72). There are two possible reasons why the keyword “PIB (amyloid PET tracer)” appears in the field of MRI studies in AD. One of which is some studies have evaluated AD using both PET-CT and MRI for pairwise comparison (72). Another potential reason is that partial studies have used PET-MR, a novel imaging technique, to evaluate AD (73).

Genetic data from the ADNI database and from transgenic model experiments have been crucial in advancing the understanding of AD pathophysiology. All of these human genetic data can be obtained from apolipoprotein E (APOE) genotyping, genome-wide association studies (GWAS), and whole-exome and whole-genome sequencing. Researchers have strived to identify new genetic susceptibility loci for AD by integrating genetic and imaging data obtained from the ADNI database. The first GWAS of an ADNI quantitative phenotype was published in 2009 (74). Potkin identified a number of loci potentially associated with hippocampal atrophy through an imaging-genetics approach, and progress has been rapid since then. Furney discovered PICALM, a significant gene associated with entorhinal cortical thickness (75). To date, GWASs have identified more than 20 genetic loci associated with the risk of AD (1, 27, 76). It is well known that APOE ε4 is the major genetic risk factor for late-onset or sporadic AD (77), whereas mutations in amyloid precursor protein (APP), presenilin-1 (PSEN1), and presenilin-2 (PSEN2) can cause early-onset or familial AD (78, 79). It is widely reported that APOE e4 allele is the strongest genetic risk factor of ARIA incidence in anti-Aβ monoclonal antibody-treated AD patients (6, 7, 80). Furthermore, transgenic murine model experiments have revealed the underlying mechanisms as well as treatment of AD in animal models using high-field MRI (22, 28–31, 81). Burgess (28) demonstrated that repeated MR imaging-guided focused ultrasound treatments led to spatial memory improvement in a Tg mouse model of AD, which may be mediated by decreased amyloid pathologic abnormalities and increased neuronal plasticity. Snow (22) demonstrated for the first time that diffusion tensor imaging (DTI) abnormalities were present in the gray matter of an AD mouse model in which both pathological hallmarks are present. Shah (31) identified that hypersynchrony of function connection (FC) may be used as a new non-invasive read-out of early AD and can be recovered by anti-Aβ treatment in TG2576 mice, providing an early therapeutic window before amyloid plaque deposition. Overall, MRI is more widely used in clinical settings for assisting in the early diagnosis and prognosis prediction of AD patients, while preclinical research toward precision medicine and pathological mechanisms in small animal models using MRI is relatively less, because of the high requirements for MR equipment, e.g., ultra-high field MRI (often ≥7.0 T) is generally required.

Furthermore, multimodal MRI is widely used for the early diagnosis of AD. In addition to sMRI and PET-MRI, fMRI has been used to measure the activity of neurons and evaluate the functional relationships between brain regions indirectly and noninvasively. First, increased hippocampal activity has been found to be associated with increased risk for AD (82, 83). Studies have revealed reduced left CA2, 3 and dentate gyrus (CA23DG) activity in cognitively intact APOE ε4 carriers, which may suggest that reduced neural activity in hippocampal subregions may underlie the compensatory increase in extrahippocampal activity in people with a genetic risk for AD prior to the onset of cognitive deficits (84). Second, arterial spin labeling (ASL) is an important MR imaging technique used to assess cerebral blood flow (CBF) quantitatively at the tissue level by magnetically labeling inflowing blood (23). AD patients have consistently shown a reduction in CBF in a posterior parietal region, including the posterior cingulate, angular gyrus, precuneus, and superior parietal gyrus (21, 85–88). Chao reported that abnormal perfusion in the precuneus may predict conversion from MCI to AD (89). Collij demonstrated that the combination of CBF and the entorhinal cortex atrophy score can significantly increase the diagnostic ability of AD, with a prediction probability of 95% (90). The ASL pattern is remarkably similar to the pattern of hypometabolism observed with FDG PET, and both modalities have similar diagnostic performance (23, 88, 91). Third, resting-state fMRI (rs-fMRI) has become one of the most widely used neuroimaging techniques for studying brain function correlated with pathological biomarkers of AD (92, 93). Studies have detected decreased FC in the default mode network (DMN), and the posterior cingulate cortex/pecuneus is the most severely affected brain region (20). DMN is one of the most vulnerable brain networks in AD because of the earliest accumulation of Aβ (19, 20, 94, 95). The early deposition of the pathological protein Aβ in the DMN region may be related to the influence of resting-state whole-brain activity on the lymphatic clearance pathway (96), suggesting that rs-fMRI technology has the potential to be used as an imaging indicator for the early identification of AD and for guiding clinical prevention and treatment. Anti-amyloid monoclonal antibodies have raised concerns about adverse effects, particularly ARIA (5, 6, 8). ARIA includes ARIA-E (parenchymal or sulcal hyperintensities on FLAIR indicative of parenchymal edema or sulcal effusions) and ARIA-H (hypointense regions on gradient recalled-echo/T2* indicative of hemosiderin deposition) (97). Timely detection and monitor of ARIA in clinical practice is crucial.

### 4.3 Stage III

Stage III focused on the study of AI techniques in the MRI analysis of the AD brain. The research focused on “#0 tau pathology” and “#1 deep learning (DL),” and the main keywords used were subjective cognitive decline, machine learning (ML), convolutional neural network (CNN), and DL.

Recently, increasing evidence has shown that hyperphosphorylated tau protein appears earlier than Aβ does and is more closely related to cognitive impairment, which is deemed to be more sensitive for the early detection of AD (98, 99). Both sMRI and FDG-PET are commonly used for measuring tau-mediated neuronal injury. Emerging MRI modalities such as DTI and rs-fMRI have also shown great potential in capturing changes due to tau pathology (100, 101). Visual assessment (102) or quantification of the hippocampus (103) is the most frequently used biomarker for measuring tau-mediated injury in AD and has been confirmed via several autopsy studies (40). In addition, atrophy is closely associated with tau accumulation in certain brain regions and is related to biomarkers of tau accumulation and brain hypometabolism, such as CSF p-tau/t-tau levels, FDG-PET, or PET imaging with tau ligands (104, 105). At present, most of the studies on tau PET have focused on specific regions of interest, including the occipital lobe, parietal lobe, temporal lobe cortex and parietal lobe cortex, in the AD cohort, which may reflect the correlation between tau protein deposition and cognitive decline (106). Ossenkoppele reported that tau PET was a promising prognostic tool for predicting cognitive decline in preclinical and prodromal stages of AD, that was superior to amyloid PET and MRI (107). Although tau PET imaging has attracted much attention compared with Aβ PET imaging, it still needs to be verified on a large scale to become a reliable tool for AD diagnosis.

In the past 6 years, the keywords have gradually evolved to use AI-powered technologies, particularly ML and DL, combined with MRI to achieve early diagnosis and prognosis of AD (108, 109). DL is an important branch of ML that uses neural networks of simple interconnected units to extract patterns from data to solve complex problems (110). A neural network is the basis of the DL method. Common AI algorithms include logistic regression (LR), support vector machines (SVMs), random forests (RFs), CNNs, and nonconvolutional artificial neural networks (NC-ANNs). ML and DL have a wide range of applications for the early differentiation of normal cognition and AD (110–112). To date, the most widely used image analysis model is CNN (113). CNNs have gained popularity quickly in MRI analysis for AD, first in 2017, with promising performance (114). Ashyam applied DeepBrainNet, a recent DL algorithm with transfer learning, to discriminate neurologic diseases on the basis of MRI brain age and achieved 86% accuracy in differentiating AD patients from healthy controls (HCs) (115). The CNN studies that combined T1WI with FDG-PET data yielded considerably different results (98% vs. 90% accuracy for AD patients vs. HCs) (116). Frizzell demonstrated that CNNs have better performance metrics than the other major algorithm types do in the classification of AD, MCI, and normal aging and the prediction of MCI conversion to AD (111). Sima applied an DL-based assistive software that can automatically detect and quantify ARIA on brain MRI scans, which has the potential to be a clinically important tool to improve safety monitoring and management of patients with AD treated with Aβ-directed monoclonal antibody therapies (117). With the development of AI, more noninvasive examinations, minimally invasive diagnoses, and more safer treatment methods have been developed, suggesting that the application of AI in AD has attracted the attention of many researchers and will be a research hotspot in the next few years.

## 5 Limitation

Some limitations of this study must be highlighted. First, all publications included in this study were downloaded from the WoSCC; therefore, they may not represent the complete research field of MRI in AD. Second, our study included only English literature, which may have led to language bias and the consequent omission of high-quality literature from other languages. Third, the publication number of countries, institutions and authors are based on all co-authors, rather than the institutions and countries of the first author. And some authors not only list the school of medicine as their affiliation but also list the hospital as a second affiliation. This led to overlap on the results, causing a bias of the influence. Finally, it takes time for articles to reach a certain number of citations after publication, which may mean that high-quality articles published in recent years have not yet reached the level of citations commensurate with their quality and may have led to research bias.

## 6 Conclusion

MRI research on AD is rapidly progressing. Advances in basic science and molecular diagnostics have provided unprecedented possibilities for early diagnosis and prognosis prediction. The United States has always been in a leading position in this field. In addition, notably, the research focus in this field has gradually shifted from invasive diagnosis and treatment to noninvasive diagnosis and more accurate safer treatment through AI-aided technologies such as ML and DL.

Recent advancements in AD treatment have focused on eliminating Aβ plaques, whereas MR imaging abnormalities collectively referred to as ARIA have been reported for several agents, thereby lowering the expectations around the eagerly awaited first-generation monoclonal antibodies. Therefore, application of the AI-aided technology to monitor and predict the efficacy and the probable side effect of new drugs or new therapies on brain MRI scans from AD patients is projected to be the research hotspot in the future.

## Data Availability

The raw data supporting the conclusions of this article will be made available by the authors, without undue reservation.
