# Correction: The global research of magnetic resonance imaging in Alzheimer's disease: a bibliometric analysis from 2004 to 2023

**DOI:** 10.3389/fneur.2025.1682998

**Published:** 2025-08-26

**Authors:** Xiaoyu Sun, Jianghua Zhu, Ruowei Li, Yun Peng, Lianggeng Gong

**Affiliations:** ^1^Department of Radiology, The Second Affiliated Hospital, Jiangxi Medical College, Nanchang University, Nanchang, China; ^2^Jiangxi Provincial Key Laboratory of Intelligent Medical Imaging, Nanchang, China

**Keywords:** Alzheimer's disease, magnetic resonance imaging, bibliometric, VOSviewer, CiteSpace

In the published article, there was an error in the Funding statement. The funding statement was displayed as “This study was supported by the youth research project of Jiangxi science and technology department (20171BAB215049)”, the funding support from the Youth Project of the Education Department of Jiangxi Province (Grant No. GJJ160259) was inadvertently omitted.

The correct Funding statement appears below:

“The author(s) declare that financial support was received for the research and/or publication of this article. This study was supported by the Youth Project of the Education Department of Jiangxi Province (Grant No. GJJ160259), as well as by the Youth Project of Jiangxi Science and Technology Department (Grant No. 20171BAB215049).”

The original article has been updated.

